# Resolving unintended pregnancy crisis: Is adoption a viable option? A cross-sectional study in Kumasi, Ghana

**DOI:** 10.1177/2050312120959181

**Published:** 2020-09-18

**Authors:** Evans Kofi Agbeno, Joseph Osarfo, Anthony Amanfo Ofori, Emmanuel Kusi Achampong, Betty Akua Oparebea Anane-Fenin, Wisdom Klutse Azanu, Kwadwo Sarbeng, Emmanuel Senanu Komla Morhe

**Affiliations:** 1 Department of Obstetrics and Gynaecology, School of Medical Sciences, University of Cape Coast, Cape Coast, Ghana; 2 Asante Mampong Municipal Hospital, Ghana Health Service, Asante Mampong, Ghana; 3 Department of Medical Education and IT, School of Medical Sciences, University of Cape Coast, Cape Coast, Ghana; 4 Department of Obstetrics and Gynaecology, School of Medicine, University of Health and Allied Sciences, Ho, Ghana; 5 Obstetrics and Gynaecology Directorate, Komfo Anokye Teaching Hospital, Kumasi, Ghana

**Keywords:** Unintended pregnancy, resolving options, child adoption, abortion, Ghana

## Abstract

**Objectives:**

Unintended pregnancy presents a crisis situation with limited options for resolution. Abortion appears to be a commonly chosen option but is stigmatized in many societies including Ghana. Keeping a child from an unintended pregnancy is also unsuitable for many people. Carrying through with the pregnancy and placing the child up for adoption is a potential management option but there is scanty literature on how viable this option is to women globally including Ghana. The study sought to assess acceptability of this option and its barriers and facilitators in Ghana.

**Methods:**

This study was a part of a bigger analytical cross-sectional study on unintended pregnancy in Kumasi conducted in three centres from January to April 2014. Exit interviews were conducted for 461 consenting women to capture data on demography, reproductive profile and acceptability of giving up a child from an unintended pregnancy for adoption. Frequencies, proportions and means were computed and presented in tables.

**Results:**

Over 85% of respondents would not give up their children for adoption as a way to manage their unintended pregnancy, whereas about 6% were undecided. A need for the child to grow up in a two-parent home was considered more important than the financial security of the adoptive parents while disappointment from family and friends came up as marked barrier to adoption.

**Conclusions:**

Keeping a pregnancy and placing the child up for adoption is presently not ideal for managing an unintended pregnancy crisis. More education is needed to increase awareness of adoption as an option in resolving this crisis while continued efforts are made at primary prevention through using contraceptives. The complex adoption process must be made friendly for women with unintended pregnancies who neither desire parenting nor abortion.

## Introduction

Unintended pregnancies present a crisis situation socially, economically and psychologically to most individuals especially young women as they are faced with limited options for resolving it. These include abortion, adoption and self-parenting.^1–6^ While induced abortions appear to be more commonly employed mostly by young or teenage pregnant women, their use as an option is debatable as there is associated stigmatization in many societies.^5,6^ Keeping a child from an unintended pregnancy may also be unsuitable for many people. Another route, less explored, is carrying the pregnancy to term and giving up the child for adoption.^1,4,5,7^

Placing a child up for adoption is the transfer through the legal system of all the parental rights that a biological parent has to a child, along with an assumption by the adopting parents of those same rights. These rights and responsibilities include the care and supervision of the child; its nurturing and training; and its physical, emotional and financial support.^7^ Studies report that adoption is generally frowned upon in most parts of the world^3,4,6–14^ even though the 1989 United Nations Convention on the Rights of the Child recognizes adoption as a form of alternative care for children who are unable to remain in their family environment.^15,16^

The situation may not be very different for developed countries. In a US study involving 21 White, unmarried pregnant adolescents and their significant others who were interviewed to determine the meaning adoption had for them, their willingness to consider adoption was found to be based on societal sanctions, low level of knowledge, anticipated psychological discomfort, and lack of support from professionals placed to offer help. It was concluded that parenthood reduces opportunities and optimal outcomes for both adolescent mother and child for which reason pregnant teenagers rarely consider adoption.^17^ Similarly, only 5.9% of those who became pregnant, in a 3-year longitudinal survey assessing the prevalence and incidence of rape and related physical and mental health outcomes among 4008 adult American women, placed their infants for adoption compared to 50% who chose to abort and 32.2% who opted to keep their infants.^9^

In spite of the fact that the major religious groupings in the world generally accept child adoption in doctrine,^18,19^ many studies, including those among West African women, on how adoption could be used to resolve infertility (not for resolving unintended pregnancy crises) showed low acceptance for child adoption.^8,14,16,20–24^ In contrast, a Ghanaian study reported that interest was shown for adopting children from an urban orphanage but one of the main difficulties encountered was unavailability of children meeting the preferences of adopters.^15^

Literature search did not reveal any African study on the acceptability of keeping an unintended pregnancy, giving birth and putting the child up for adoption as an unintended pregnancy management option. Fostering comes close to adoption in West Africa. With fostering, children are sent away to be raised by relatives and nonrelatives among many ethnic groups.^25^ Child fostering is, indeed, an age-old mechanism in sub-Saharan Africa to care for children who would have otherwise been left on their own after the death of parents or when biological parents shirk their responsibility towards their children.^26^ Literature search did not reveal any study linking fostering to resolving unintended pregnancy crisis.

In Ghana, adoption is lawful and the Children’s Act (560) of 1998 spells out the procedure. The process is generally thought of as being tedious and the focus has been on adopting from orphanages mostly. This law, however, is silent on how a woman carrying an unintended pregnancy can directly give the baby up for adoption either during the pregnancy or immediately after delivery.^27^ Abortion in Ghana is generally stigmatized. Also, keeping an unintended pregnancy and self-parenting is not suitable for some people.^28,29^ Carrying through with the pregnancy and later placing the child up for adoption is an option which could help resolve such dilemma but there is very little literature on how viable this option is to women throughout the world and Ghana in particular. Some noted barriers to adoption consideration are lack of knowledge about the process of adoption,^15,17,30^ stigma^10^ and the reaction of family and friends.^5^ This study assessed the acceptability of keeping and placing a child up for adoption as a viable option in resolving unintended pregnancy crisis and its barriers and facilitators.

## Methodology

### Study area, design and procedures

This study was part of a larger study looking at various aspects of unintended pregnancies and employed an analytical cross-sectional design. It was conducted at three facilities. Two of the facilities belonged to Marie Stopes International Ghana and were located at Adum and Alabar in the Kumasi Metropolis. The third facility was a Ghana Health Service facility Maternal and Child Health Hospital (MCHH) also located in the Kumasi Metropolis. The study was conducted between 1 January 2014 and 30 April 2014. The study facilities offer comprehensive abortion care services in addition to full or partial traditional antenatal care services and are described in greater detail in previous works emerging from the larger study.^31,32^

The study team trained health staff at the study facilities on what constituted an unintended pregnancy. This was to help them identify potential participants. A pregnancy was categorized as unintended if it was perceived by the woman to be undesired or mistimed at the time of realization of conception. Mistimed, as used in this context, means the pregnancy was desired but deemed to have occurred sooner than expected at the time of realization of conception. Women with unintended pregnancies constituted the study population and were included if (1) they were confirmed pregnant and were visiting the MSIG and MCHH centres for the first time irrespective of gestational age and (2) they gave consent to be interviewed for the study. The exclusion criteria were (1) refusal to give consent and (2) if they had complications of induced abortions with unstable clinical states at presentation. Some of the women in the latter group were recruited into the study at a much later time after they had recovered fully. The women were pre-informed of an interview that was to be conducted after they had completed their medical consultation and were about to exit the facilities. All consenting women were invited into the study daily until the desired sample size was obtained. Pregnancies were confirmed either by serum or urine pregnancy test and/or pelvic ultrasonography.

Trained research assistants administered a questionnaire in the local Asante Twi language as it was known from previous engagements with the centres that most of the patrons had limited English reading and comprehension skills. The first section of the questionnaire covered demographic and reproductive profiles including age, level of education, parity and gestational age. The second section focused on the acceptability of carrying an unintended pregnancy to term, delivering and giving the child up for adoption and also assessed facilitators and barriers to this option (see Supplementary File for questionnaire). The questionnaire was developed de novo based on literature review of the topic in question and personal experiences with clients gathered over many years of clinical practice. The questionnaire was pre-tested at the Manhyia Hospital, also in Kumasi and appropriate changes made to its structure and translation. Consent was verbal and it was explained to the study women that they could stop the interview at any stage and not have any health services withdrawn from them.

### Sample size calculation

The sample size was calculated using the formula N = pqz^2^/d^2^

Where N = sample size, z = level of confidence at 95% = 1.96; d = allowable error = 0.05; p = proportion of unintended pregnancies and q = 1 − p.

The proportion of unintended pregnancies in Ghana according to the 2008 demographic and health survey was 40%.

N was calculated as approximately 369. A 25% adjustment was made for non-response which finally brought the sample size to 461. The 25% non-response rate was assumed because of the sensitive nature of the subject and the probability of many respondents opting out in the course of the interview as observed during the pretesting.

### Data management and analysis

Completed questionnaires were checked for accuracy and consistency and the data double-entered in Epi Info 7 (7.1.1.14), cleaned and analysed using same. Means, ranges, frequencies and proportions were computed and presented in tables.

### Ethical considerations

Ethical approval for the study was granted by the Committee of Human Research, Publications and Ethics, Kwame Nkrumah University of Science and Technology (Ref: CHRPE/AP/032/14). Written permissions were obtained from the management of MSIG (REF: MSIG/HMDT/2013) and MCHH (Ref: KM/AD-I/13/10). The study was registered with the Komfo Anokye Teaching Hospital Research and Development Unit (Ref: RD/CR13/161). Strict confidentiality of participants’ identity was assured since no names were used.

## Results

Data from 442 women were analysed. Nineteen women were later determined to have been incorrectly categorized as carrying unintended pregnancies and were excluded from analysis. Participants’ ages ranged from 13 to 46 years with a mean (standard deviation (SD)) of 29 years (5.5). About 12% (53/442) of respondents were under 20 years of age while less than half (198/442) had senior high/technical/vocational school education. The majority of the respondents had never been married (59.8%, 262/442). Table 1 shows the demographic characteristics of the respondents, while Table 2 shows their reproductive profiles. The number of pregnancies (gravidity) ranged from 1 to 21 with a median of 2, while the number of births ranged from 0 to 9 and also had a median of 2. The gestational age ranged from 4 to 39 weeks with a mean (SD) of 16 weeks (10.7) and about half were in the first trimester (50.4%, 178/442). Less than a third of the respondents had had a previous induced abortion.

**Table 1.** Sociodemographic characteristics of respondents.

Variable	Frequency	Percentage
Facility of recruitment		
MSIG	215	48.6
MCHH	227	51.4
Age (years)		
<20	53	12.0
⩾20	389	88.0
Economic status		
Income earners^a^	355	80.3
Non-income earners	87	19.7
Marital status		
Currently or ever married	176	40.2
Never married	262	59.8
Educational level		
⩽Basic^b^	244	55.2
>Basic	198	44.8
Religion		
Christian	392	89.3
Muslim	47	10.7

MCHH: maternal and child health hospital; MSIG: Marie Stopes International, Ghana.

With the exception of religion for which the respondents were 439, all variables had 442 respondents.

a Of the income earners, 67.3% (239/355) earned an average monthly income of ⩽US$60 which was the equivalent of the national minimum wage at the time of the study while the rest earned higher.

b Basic education refers to schooling up to Junior High School level (at least 9 years of schooling in total).

**Table 2.** Reproductive profile of respondents.

Variable	Frequency	Percentage
Gravidity		
1	188	42.5
⩾2	254	57.5
Parity		
0	232	52.5
⩾1	210	47.5
Gestational age (weeks)		
⩽13	178	50.4
<13	175	49.6
Previously induced abortion		
Yes	124	28.1
No	318	71.9
Pregnancy intention at conception^a^		
Unwanted	44	10.5
Mistimed^b^	377	89.5

a Refers to pregnancy intention when the woman realized she was pregnant.

For gravidity, parity and previous induced abortion, the respondents were 442. Respondents for gestational age were 353 while those for pregnancy intention at conception were 421. ^b^Mistimed as used here means the pregnancy occurred earlier than desired as at the time of conception.

Over 85% (381/442) of respondents did not want the adoption option as a means of resolving the crisis of an unintended pregnancy. About 8% (35/442) were willing to do consider the adoption option, whereas 5.9% (26/442) were undecided. Regarding the actual decisions taken to resolve the current unintended pregnancy, less than 1% (0.01%, 4/442) of the women opted to deliver and give the child up for adoption, while 55.4% (254/442) decided on self-parenting with 47.3% (193/442) opting for termination of their pregnancies.

Table 3 shows factors that could act as barriers and facilitators to considerations of adoption by women with unintended pregnancies. More than half of the respondents (59.1%, 215/364) felt a child needs to grow in a two-parent home and that this would make them consider giving up their infants for adoption in any future unintended pregnancy. This was followed by about a quarter of respondents who felt not being ready to be a parent would drive them to consider adoption in future unintended pregnancies. Beliefs about parental responsibility owed to the child and a lack of knowledge about the adoption process came up as prominent barriers to future considerations for accepting adoption in situations of unintended pregnancies.

**Table 3.** Facilitators and barriers influencing consideration for putting up a child from an unintended pregnancy for adoption.

Variable	Frequency	Percent
What would influence you to consider making an adoption plan in any future unintended pregnancy (ies)? (n = 364)		
Child needs two-parent home	215	59.1
If the adoptive parents are financially secured	33	9.1
Do not feel ready to parent	94	25.8
Parenting would interfere with educational goals	22	6.0
General factors against making adoption plans (n = 405)		
Beliefs about parental responsibility	244	60.2
Concerns about peer rejection	29	7.2
Inability to locate resources	22	5.4
Lack of knowledge about the adoption process	74	18.3
Other (social stigma; shame, complex adoption process; cumbersome legal process, socio-cultural beliefs, emotional attachment to the child)	36	8.9
Family and friends’ reaction to making adoption plans (n = 405)		
I do not expect any reaction since they do not care about what I do with my life	7	1.7
They will be disappointed in me and abandon me	243	60.0
They will be disappointed in me but will offer support	123	30.4
They will be happy and support me	10	2.5
Other (they will not support the idea but not sure of their reaction, they will prefer to take the babies up themselves and they will see me as a fool)	22	5.4

There was a general perception of likely disapproval from family and friends regarding considerations of placing a child from an unintended pregnancy up for adoption and 90.4% (366/405) of respondents felt family and friends would be disappointed in them if they were to consider giving up their babies for adoption because their pregnancies were unintended. Of these, 66.4% (243/366) felt their family/friends would also abandon them in addition to being disappointed, while the rest (123/366) thought family/friends would still offer their support despite being disappointed with them. It is noteworthy that only less than 2% (7/405) of respondents felt they did not expect any reaction from family and friends since they did not perceive their lives were aligned with that of any family/friends.

Only a quarter of respondents (25.5%, 106/416) said they had previously heard of adoption being discussed on radio or television, while 43.6% (181/415) had heard of it from their religious organizations (data not shown in tables).

## Discussion

The study assessed the acceptability of keeping an unintended pregnancy and putting up the child for adoption as an option for resolving unintended pregnancy crisis and over 85% of respondents were not in favour of this option. Major barriers identified were beliefs about parental responsibility owed to the child, a lack of knowledge about the adoption process, and the fear of how their family and friends’ reaction would be. Many studies have explored the use of child adoption for solving infertility challenges in Africa.^8,9,12–14,19,21,22,33^ Previous studies have also shown similar high unacceptance rates. However, to the best of our knowledge, the current study is the first to assess the potential for child adoption as a means of resolving unintended pregnancy crisis in Africa. The major findings have implications for strategies needed to improve acceptability of putting up a child from an unintended pregnancy for adoption in Ghana and possibly beyond in other African settings.

The aforementioned barriers were similar to other works published in terms of barriers to adoption in general.^8,9,12–14^ However, barriers specific to keeping unintended pregnancy and giving the child up for adoption in Africa were not found in our search and thus further buttressing the novelty of the current study.

A major barrier to considering adoption was the beliefs about parental responsibility. These beliefs border on a duty of care to the child. This is further reinforced by the mother–child bond and is easy to appreciate why this would be a barrier to completely handing over all legal rights to one’s child from an unintended pregnancy to adopters. This sense of parental responsibility is more or less expected by the larger society so that shirking it by placing a child up for adoption is likely to be met with societal disapproval. Assessing the community’s perspective on placing a child up for adoption as a means of managing an unintended pregnancy was beyond the scope of this work. However, one may draw reasonable conclusions that it is likely to be unfavourable based on the response of anticipated disappointment from family and friends given by over 90% of the respondents. The role of family and friends in considerations of a sensitive nature such as giving a child up for adoption derives from the communal nature of Ghanaians and most African communities. This same communal nature underpins many foster parent–child relationships. Each individual belongs to a community of people or family^20,33^ who have a stake in major life decisions^2,24^ and reflects in what the Zulu tribe from South Africa refer to as ‘ubuntu’ (I am because you are). The role of family and friends is recognized and far-reaching and reflects in the observation that only a few respondents felt they could not be bothered by the reaction of their family and friends if they were to consider keeping their unintended pregnancies and giving up the child for adoption. From the experience of the authors, one is not guaranteed financial support from family and friends if one chooses to keep an unintended pregnancy instead of giving up the child for adoption. In spite of this, family and friends and perhaps the community as a whole constitute major stakeholders and should not be left out of educational programmes aimed at increasing acceptability of keeping unintended pregnancies to term and placing the resulting child up for adoption. Other specific barriers to adoption plans given were social stigma, shame, complex adoption processes and cumbersome legal processes. These were similar to those found in other studies even though they were not the major barriers in our study.^8,25–28^

Contrary to popular belief, the financial security of the adoptive parents ranked a distant third among the facilitators for keeping an unintended pregnancy and placing the child up for adoption. Instead, the need for the child to be in a two-parent home, which would be difficult for the biological mother to provide, came up more strongly as a facilitator. This observation carries implications for packaging information on child adoption as an option for managing unintended pregnancy crisis. It appears that, beyond a minimum care, emphasizing the potential financial security a child is likely to get from rich adoptive parents could be unproductive. Close to a third of respondents in the present study either did not feel ready to parent or thought parenting would interfere with their educational goals. The former may be related to low economic power with reported average monthly incomes of US$60 or below for the majority of respondents but this is unlikely to be the case since an overwhelming majority rejected the possibility of giving up their infants for adoption despite their low earnings.

The concept of ‘unintended pregnancies’ is often associated with teenagers or young single women but the findings show that it can occur in older married women as well. This brings to mind the need to increase awareness and uptake of family planning methods as the vast majority of respondents felt their unintended pregnancies were desirable but wrongly timed compared to the few who declared their pregnancies strictly unwanted at the time of realization of conception.

The perspective of prospective adopters was beyond the scope of this study, but some Ghanaian studies have reported conflicting observations. While one study found a reasonable demand for adopted children from an orphanage in the capital city,^15^ another hospital-based study in rural northern Ghana found child adoption psychologically dissatisfying to infertile women.^23^ The reason for the different perspectives is unclear but differences in education, income levels and beliefs may play a role. In the former study, it was noted that prospective adopting parents often preferred infants or very young children but these were not always available.

The study is limited by the choice of a cross-sectional design which restricts deeper exploration of issues and responses selected. A qualitative approach using focus group discussions and/or in-depth interviews would have allowed for broader exploration of underlying issues rather than the ‘straight jacket’ approach used here. Nonetheless, the findings presented are relevant as they improve our understanding of many aspects of the sociology of unintended pregnancies and give useful pointers for future research. Another limitation is the failure to examine the perspectives of potential adopters around adoption. It would be useful to see if they are consistent with the perspectives of the women with unintended pregnancies. Further in-depth qualitative studies are needed to explore nuances around the observed barriers to adoption as well as the perspectives of potential adopters and community norms around adoption.

## Conclusion

Keeping unintended pregnancies and giving the child up for adoption is not a popular option for resolving unintended pregnancy crises. In addition to the noted barriers, it is possible that this finding emanates from very little or no awareness of adoption as a strategy for managing unintended pregnancy. Stakeholders in the child adoption space, especially the social welfare department, need to take up the challenge to push the agenda through sustained information, education and communication strategies. Religious outfits must be encouraged to be less judgemental of teenagers and single women who get pregnant and rather use their platform to encourage carrying unintended pregnancies to term for adoption since termination is frowned upon by the religious groupings. This has the potential of reducing unsafe abortions in the system and its attendant maternal mortalities. Furthermore, this strategy will help reduce the incidence of mothers abandoning newborn babies as is often observed. The National Population Council is also expected to do more to increase the acceptability of family planning methods to help reduce the burden of mistimed pregnancies.

## Supplemental Material

SUPPLEMENTARY_FILE_questionnaire_1 – Supplemental material for Resolving unintended pregnancy crisis: Is adoption a viable option? A cross-sectional study in Kumasi, Ghana Click here for additional data file. Supplemental material, SUPPLEMENTARY_FILE_questionnaire_1 for Resolving unintended pregnancy crisis: Is adoption a viable option? A cross-sectional study in Kumasi, Ghana by Evans Kofi Agbeno, Joseph Osarfo, Anthony Amanfo Ofori, Emmanuel Kusi Achampong, Betty Akua Oparebea Anane-Fenin, Wisdom Klutse Azanu, Kwadwo Sarbeng and Emmanuel Senanu Komla Morhe in SAGE Open Medicine
