# Supplementary material for: Resolving unintended pregnancy crisis: Is adoption a viable option? A cross-sectional study in Kumasi, Ghana
Source: SAGE Open Med. 2020 Sep 18;8:2050312120959181. doi: 10.1177/2050312120959181 (PMC7506785; doi:10.1177/2050312120959181)
Supplement: SUPPLEMENTARY_FILE_questionnaire_1 – Supplemental material for Resolving unintended pregnancy crisis: Is adoption a viable option? A cross-sectional study in Kumasi, Ghana [file SUPPLEMENTARY_FILE_questionnaire_1.pdf]

SOCIO-DEMOGRAPHIC CHARACTERISTICS/REPRODUCTIVE PROFILE

No:	QUESTIONS AND FILTERS	RESPONSES	CODE	SKIPS
1	Initials			
2	Age(yr)			
3	Telephone no. (just for follow up research)			
4	Partner's telephone no.(purely for follow up research)			
5	Occupation	Unemployed<br>Student<br>artisan (hairdressing, dressmaking etc)<br>trader/businessman<br>civil/public servant<br>teacher<br>farmer<br>health worker<br>banker<br>other (specify)	1<br>2<br>3<br>4<br>5<br>6<br>7<br>8<br>9<br>10	
6	Religion	Christian<br>Moslem<br>spiritualist/traditionalist<br>other (specify)	1<br>2<br>3<br>4	
7	Marital status	Married<br>.single<br>.cohabiting<br>divorced/separated<br>widowed	1<br>2<br>3<br>4<br>5	

8	Educational status	None<br>Primary<br>.middle/JHS<br>SHS<br>Tertiary<br>other(specify)	1<br>2<br>3<br>4<br>5<br>6	
9	Average monthly income (GH₵):	Nil<br>0-159<br>160-499<br>500-999<br>1000-1999<br>≥2000	1<br>2<br>3<br>4<br>5<br>6	
10	Gravidity			
11	Parity			
12	Previous abortions	Spontaneous<br>.Induced	1<br>2	
13	Gestational age of current pregnancy			
14	Intension for the pregnancy at conception	did you want to wait until later(mistimed)?<br>did you not want to have any (more) children at all(unwanted)	1<br>2	

ACCEPTABILITY OF ADOPTION

33	What do you know about adoption and making an adoption plan?			
34	What do you think about adoption and making an adoption plan?			
	After your encounter at this facility, have you considered adoption as an	**Yes**<br>**No**	**1**<br>**2**	**If no or undecided, skip to 36**

	option for resolving this unintended pregnancy?	**undecided**	**3**	
35	What is the most important factor which influenced you in considering/making an adoption plan for this pregnancy?	Do not feel ready to parent<br>Decision in best interest of child for financial reasons<br>Child needs two parent home<br>Parenting would interfere with educational goals<br>other(specify)……	1<br>2<br>3<br>4<br>5	
36	What do you think influenced you **<u>NOT</u>** to consider making an adoption plan or making you undecided?	Lack of knowledge about the adoption process<br>Inability to locate resources<br>Beliefs about parental responsibility<br>Concerns about peer rejection<br>Other: specify-<br>-Social stigma<br>-Shame<br>-cumbersome legal process<br>-complex adoption procedure<br>-anymore?…..	1<br>2<br>3<br>4<br>5<br>A<br>B<br>C<br>D<br>e	
37	What is the most important factor which will influence you in considering/making an adoption plan for any future unintended pregnancy?	Do not feel ready to parent<br>Decision in best interest of child for financial reasons<br>Child needs two parent home<br>Parenting would interfere with educational goals<br>other(specify)……	1<br>2<br>3<br>4<br>5	
38	What do you think will be the reaction of your family/friends if you made	I don't expect any reaction since they do not care about what I do with my life<br>They will be disappointed in me and abandon me	1<br>2	

	an adoption plan for this pregnancy?	They will be disappointed in me but will offer support<br>They will be happy and support me<br>Other (they will not support the idea but not sure of their reaction, they will prefer to take the babies up themselves and they will see me as a fool)	3<br>4<br>5	
39	Were you ever involved in any discussion on adoption prior to becoming pregnant?	Yes<br>No	1<br>2	
40	Do you have any experiences with adoption?	Yes<br>No	1<br>2	
41	If yes, what is it?	Personal<br>family member<br>friend<br>other (specify)	1<br>2<br>3<br>4	
42	Did you learn/hear about adoption in school?	Yes<br>No<br>Not applicable (never being to school)	1<br>2<br>3	
43	Have you ever heard about adoption being discussed in the religious organization you belong to?	Yes<br>No<br>Not applicable (for atheist)	1<br>2<br>3	
44	Have you heard about the subject of adoption being discussed/mentioned in the media?	Yes<br>No	1<br>2	
